# Evaluation of the relationship between types of menopause and cognitive function

**DOI:** 10.55730/1300-0144.5918

**Published:** 2024-07-24

**Authors:** Meltem DURAKLI ULUKÖK, Ufuk ATLIHAN, Onur YAVUZ

**Affiliations:** 1 Department of Neurology, Private Çankaya Medical Center, İzmir, Turkiye; 2 Department of Gynecology and Obstetrics, Private Karataş Hospital, İzmir, Turkiye; 3 Department of Gynecology and Obstetrics, Faculty of Medicine, Dokuz Eylül University, İzmir, Turkiye

**Keywords:** Age at menopause, cognitive decline, cognitive performance, early menopause

## Abstract

**Background/aim:**

Menopause is often accompanied by neurological symptoms, including cognitive difficulties, especially with memory and attention. The purpose of the present study was to examine the relationship between the timing of menopause and the cognitive performance of menopausal patients who applied to our neurology clinic with complaints of forgetfulness.

**Materials and methods:**

The data of 538 women who applied to the neurology clinic with complaints of forgetfulness between January 2018 and January 2024 and underwent neuropsychological evaluations were scanned retrospectively. A total of 96 patients who applied to the neurology and menopause clinics were included in the study.

**Results:**

The attention orientation, verbal fluency, memory, and total Addenbrooke’s cognitive evaluation battery-revised (ACE-R) test scores were significantly higher in the >50-year-old menopausal group when compared to the other groups (p < 0.001). A statistically significant, negative, and weak relationship was detected between the body mass index (BMI) and memory scores (r = 0.3, p < 0.001). A statistically significant, positive, and weak relationship was detected between the high-density lipoprotein level and verbal fluency score, memory score and ACE-R score (r = 0.3, p < 0.001). A statistically significant, negative, and weak relationship was detected between the BMI and memory scores (r = 0.3, p < 0.001).

**Conclusion:**

The relationship between the cognitive performance of menopausal women and the timing of menopause was examined herein. The ACE-R, attention and orientation, visual, verbal fluency, and memory scores were significantly higher in women ≥50 years of age who entered menopause compared to those <40 years of age and those between 40 and 50 years of age. This study provides evidence that advancing age at menopause is associated with better cognitive performance. This relationship is most evident in the areas of the attention, verbal fluency, memory, and visuospatial domains.

## 1. Introduction

The average age for menopause is 49–52 years, and the onset of menopause indicates the end of the reproductive period [1]. Aside from the average age, when menopause begins before the age of 45, it is referred to as early menopause, and when it begins at or before the age of 40, it is known as premature ovarian insufficiency (POI). The prevalence of those two kinds of menopause is predicted to be approximately 5% and 2%, respectively [2,3]. Induced menopause refers to the permanent cessation of menstruation because of surgical ovarian excision or ovarian ablation with chemotherapy or radiation [4]. Early menopause and POI are generally associated with several elements that might contribute to follicular depletion beyond gonadal hormone levels, the most crucial of which is family background, which increases the risks by approximately twelve times [5,6]. The following factors are linked to early menopause and POI: smoking, body mass index (BMI) score <18.5 kg/m^2^, and low socioeconomic status [7,8]. While natural menopause causes endogenous estrogen levels to gradually decrease, surgical removal of one or both ovaries may produce an earlier and occasionally more abrupt decline in estrogen levels [9]. Estrogen plays important roles in adulthood, not only in the menstrual cycle, but also in the brain due to its neuroprotective, neurotrophic, and antioxidant effects [10]. Estrogen has been shown in earlier experiments to promote brain mitochondrial survival, improve synaptic plasticity, and strengthen neuroprotective effects [11]. As a result of these effects, neurological diseases may be exacerbated by estrogen deficiency. Menopause frequently comes with neurological side effects, such as memory and focus problems and cognitive impairments [12]. Nevertheless, the long-term association between cognitive abilities and the postmenopausal era remains unclear. The relationship between menopausal hormone replacement therapy (HRT) and age at menopause with the likelihood of dementia, cognitive impairment, or cognitive performance in later life is not well supported by the available data [12,13]. The estrogen receptor network, which is one of the main regulatory systems of the brain, plays a very important role in cognitive disabilities. Important estrogen receptors are found in areas that are essential for learning and memory, such as the hippocampus, amygdala, posterior cingulate cortex, and prefrontal cortex [10,14]. Neuronal circuit function, intracellular signaling, and the availability of energy in brain neurons may all be impacted by estrogen or its receptor network [15]. Negative neurological consequences linked to early menopause are mostly caused by an early and protracted decrease of ovarian-derived 17β-estradiol (E2) [16]. In addition to its effects on neurotransmitter levels and activity, estrogen enhances the development of synapses and neuronal growth, which in turn controls the second messenger system, calcium homeostasis, and antioxidant activity [17,18]. It was shown that women with early menopause are at great risk of developing type-2 diabetes mellitus (DM) when compared to those of normal menopausal age (>45 years) [19]. Mild cognitive deficits have also been linked to DM for more than ten years [20]. During the menopausal transition, various cognitive skills, such as object recognition, object placement performance, executive functioning, recall of words and proficiency, and linguistic and spatial working memory, may be impacted [21].

Longitudinal studies conducted on women not receiving HRT have shown certain decreases in verbal fluency, verbal and episodic memory, attention, and executive function [22,23]. When preoperative and postoperative cognition were compared in young women with surgical menopause, an important cognitive decline was detected (especially in the regions of general cognition and verbal memory) [24,25]. The cognitive impairment risk increases in women with surgical menopause prior to the age of 49 when compared to age-matched controls, and this risk also increases the younger they are at the time of surgery [26]. The rate of cognitive impairment increases with the age at which surgical menopause occurs (especially in regard to episodic and semantic memory) [24]. It was reported that women who go through menopause before the age of 40 have weaker visual and verbal memory than those who do not [27]. Considering different cognitive outcomes, different forms of menopause must be taken into account in the design and analysis of studies on menopause and cognition. The aim of the current research was to examine the relationship between the timing of menopause and the cognitive performance of menopausal patients who applied to our neurology clinic with complaints of forgetfulness.

## 2. Materials and methods

The present investigation had a retrospective case-control design and was conducted in line with principles of the Declaration of Helsinki. Informed voluntary consent forms were obtained from the patients. The study began after receiving ethics committee approval from the Dokuz Eylül University Ethics Committee (Date: May 29, 2024, Number: 2024/19–11). Using patient information obtained from our hospital database, the data of 538 women who applied to the neurology clinic with complaints of forgetfulness and underwent neuropsychological evaluation between January 2018 and January 2024 were retrospectively scanned. The presence of applications to the menopause clinic by the evaluated women was additionally investigated. Hence, a total of 96 patients who applied to the neurology and menopause clinics were included in the study. Those who had previously experienced neurological illness, psychological illness, had a previous history of dementia, and those with unknown menopause history were excluded from the study. The participants’ demographic characteristics, e.g., parity, education level, age, smoking and alcohol use, and age at menarche, were evaluated retrospectively. Serum biochemistry and hormonal analysis samples of the patients from the hospital database were evaluated retrospectively. Fasting blood sugar, fasting insulin, total cholesterol, high-density lipoprotein (HDL), low-DL (LDL), triglyceride, and thyroid stimulating hormone (TSH) levels of the patients were recorded. Homeostasis model evaluation (HOMA) was employed to evaluate insulin sensitivity ([fasting blood sugar mmol/mL) × fasting insulin (μmol/L)]/22.5). The menopausal age was determined as that at the last menstrual period for all the menopause types. Menopause was defined as having no menstrual period for the last year or having a TSH level >40 pg/mL in laboratory tests [28]. Smoking was defined as smoking 10 cigarettes/day according to the World Health Organization (WHO) definition [29]. Alcohol use was defined as using alcohol >3 days a week, according to data from the US National Institute on Alcohol Abuse and Alcoholism [29].

Age at menopause was referenced as a categorical variable as <40, 40–49, and ≥50 years unless otherwise stated in this study. Those under the age of 40 were included in group 1, those between the ages of 40–49 were included in group 2, and those aged 50 and over were included in group 3. From a clinical perspective, those age categories distinguished women who experienced menopause after the natural mean age (≥50 years) from those who experienced menopause at an earlier age (premature menopause). Menopause before the age of 40 was referred to as early menopause [30]. The lack of menstruation without a record of bilateral oophorectomy and/or hysterectomy was referred to as natural menopause. The term surgical menopause refers to the permanent cessation of menstruation as a result of ovarian ablation (with radiation or chemotherapy) or surgical removal of the ovaries (removing the ovaries alone or the ovaries plus fallopian tubes) [4]. The Addenbrooke’s cognitive evaluation battery-revised (ACE-R) test includes the evaluation of attention capacity, memory performance, verbal fluency, language functions, and visuospatial functions. The reports of patients who underwent ACE-R were examined for cognitive evaluation and the total ACE-R score (maximum 100) obtained from five cognitive domains scored individually for each patient (attention and orientation (0–18 points), verbal fluency (0–14), memory (0–26), language (0–26) and visual-spatial function (0–16) scores were calculated. The screening cut-off score for the ACE-R test was 87 (sensitivity of 83, specificity of 54), and the diagnostic cut-off score was taken as 83 (specificity of 80, sensitivity of 52) [31,32]. Statistical analysis was conducted using IBM SPSS Statistics for Windowsi26.0 (IBM USA). The normality of the distribution was evaluated with the Kolmogorov–Smirnov test. The analysis of normally distributed parameters was conducted with analysis of variance and posthoc tests. The chi-squared and Fisher’s exact tests were employed in the analysis of the categorical data. Quantitative data of the patients were presented as the mean ± standard deviation (SD) (minimum–maximum). Qualitative data were given as numbers (n) and percentages (%). Pearson relationship analysis was utilized in the relationship analysis between the variables. The 95%xconfidence interval (CI) was used to analyze the results. p < 0.05 was accepted as statistically significant.

## 3. Results

The age mean of the participants was 44.3 ± 6.2 years. The mean BMI score of the patients was 24.2 ± 1.5 kg/m^2^. Among the patients, 74 (77%) had natural menopause, and 22 (23%) surgical menopause. The mean BMI score in the ≥50-year-old menopausal group was 23.5 ± 1.6 kg/m^2^, which was lower than in the other groups (p < 0.001). As shown in Table 1, no significant difference was detected between the groups in terms of the education level, parity, smoking, and alcohol use.

The mean fasting glucose level was 87.3 ± 9.5 (mg/dL) in the <40-year-old menopausal group, which was significantly higher than in the other groups (p = 0.03). The mean total cholesterol level was 202.8 ± 21.5 (mg/dL) in the ≥50-year-old menopausal group, which was significantly lower than in the other groups (p < 0.001). HDL level in the ≥50-year-old menopausal group was 54.4 ± 5.9 (mg/dL) and was significantly higher than the other groups (p < 0.001). TSH level in the <40-year-old menopausal group was 1.77 ± 1.12 (mIU/L) was detected and was significantly lower than in the other groups (p = 0.04). HOMA level in the <40-year-old menopausal group was 2.3 ± 0.17 and was significantly higher than in the other groups (p = 0.005) (Table 2).

The attention orientation score was 17.3 ± 0.7 in the ≥50-year-old menopausal group, and was significantly higher than in the other groups (p < 0.001). The visual score was 15 ± 0.8 in the ≥50-year-old menopausal group, and was significantly higher than in the other groups (p < 0.001). The verbal fluency score was 12.2 ± 0.8 in the ≥50-year-old menopausal group, and was significantly higher than in the other groups (p < 0.001). The memory score in the ≥50-year-old menopausal group was 22.3 ± 0.8, and was significantly higher than in the other groups (p < 0.001). The ACE-R score in the ≥50-year-old menopausal group was 91.9 ± 1.4, and was significantly higher than in the other groups (p < 0.001) (Table 3).

A statistically significant, negative, and weak relationship was detected between the BMI and memory scores (r = 0.3, p < 0.001). A statistically significant, negative, and weak relationship was detected between the BMI and ACE-R scores (r = 0.2, p = 0.01). A statistically significant, negative, and weak relationship was detected between the total cholesterol level and attention orientation score (r = 0.3, p < 0.001). A statistically significant, negative, and weak relationship was detected between the total cholesterol level and visual score (r = 0.4, p < 0.001). A statistically significant, negative, and weak relationship was detected between the total cholesterol level and verbal fluency score (r = 0.3, p = 0.002). A statistically significant, negative, and weak relationship was detected between the total cholesterol level and memory score (r = 0.2, p = 0.02). Another statistically significant, negative, and weak relationship was detected between the total cholesterol level and ACE-R score (r = 0.4, p < 0.001). A statistically significant, negative, and weak relationship was detected between the LDL level and visual score (r = 0.3, p = 0.003). A statistically significant, negative, and weak relationship was detected between the LDL level and ACE-R score (r = 0.2, p = 0.04). A statistically significant, positive weak relationship was detected between the HDL level and verbal fluency score (r = 0.2, p = 0.004). A statistically significant, positive weak relationship was detected between the HDL level and memory score (r = 0.3, p < 0.001). Another statistically significant, positive weak relationship was detected between the HDL level and ACE-R score (r = 0.3, p < 0.001) (Table 4).

## 4. Discussion

In the current study, in which the relationship between the cognitive performance of menopausal women and the timing of menopause were examined, the ACE-R, attention and orientation, visual, verbal fluency, and memory scores were significantly higher in women who experienced menopause at ≥50 years of age than those who went through it between the ages of 40 and 50 and ≤40. Several studies have looked at women who had early menopause once it became clearer that surgically induced menopause, especially before the age of 45, had an impact on all areas of health, including cognition [26,33]. The North American Menopause Society 2014 Guideline and Endocrine Society Clinical 2015 Guideline discussed the significance of analyzing the types and context of menopause in treatment, especially in terms of cognitive complaints [34,35]. Literature findings about the correlation between cognitive function and age at menopause are contradictory. Yoo et al. [36] conducted a study on Korean women and reported that menopause at a later age was linked with a 20% lower risk of developing Alzheimer’s disease (AD). Najar et al. [37], however, reported that each year after menopause was linked with a 7% higher risk of developing AD. Yamada etial. [38] reported no significant relationships between these two variables. Needham et al. [39] did not find any differences in the age at menopause-cognition relationship according to the types of menopause. Hao et al. [40] found that earlier natural menopause (≤40 and 41–45 years) was associated with a smaller risk of developing all-cause dementia, but later menopause (51–55 and >55) was associated with a smaller risk of developing dementia. Data about the relationships between surgical menopause and the risk of developing dementia are limited. A metaanalysis that covered 12,731 women reported that surgical menopause was not correlated with the risk of developing dementia; however, surgical menopause was associated with a higher risk in the ≤45 age group [41]. Hao et al. [40] reported that surgical menopause, both before the age of 40 and after the age of 55, was linked with a high risk of developing dementia. In order to determine if early menopause increased the risk of developing all-cause dementia and its subtypes in the general female population, Liao et al. [42] carried out a large-scale investigation using UK Biobank Data, related changes in the brain structure by magnetic resonance imaging, and potential underlying mediators. Their results during a mean follow-up of 12.3 years indicated that, in comparison with women aged ≥50 years, an earlier age of menopause was linked to a higher prevalence of dementia from all causes. They also reported that the spontaneous or surgical triggering of early menopause did not make a significant difference in the risk of developing dementia. How early menopause causes dementia, and its fundamental causes remain uncertain. Bove et al. [24] quantified pathological changes in brain autopsies of 600 women in two longitudinal studies and reported that early menopause age was linked with a more rapid decrease in general detection and AD neuropathology, particularly neuritic plaques. Relationships between memory performance and menopause age might vary depending on the types of memory tasks. In the present study, women who went through menopause after the age of 50 had a verbal fluency score that was noticeably higher than that of the other two groups. Similar to the current study, Kuh et al. [43] conducted a cohort study and observed small variations in verbal memory according to the menopause type. They also reported that women who entered menopause naturally at older ages maintained a significant positive difference in verbal memory until the age of 69, but could not maintain processing speed when compared to women who entered menopause earlier. According to the findings of a study by Mitchell and Woods [44], memory-related issues, particularly trouble recalling words or numbers, were the most prevalent complaints. Italian women going through menopause have worse memory abilities, according to Betti et al. [45]. According to a study conducted by Schaafsma et al. [46], the most prevalent problems in menopausal women were attention deficit, reaction time, and verbal memory disorders. A number of cognitive examinations, including backward counting, serial seven subtraction testing, immediate and delayed 10-name recall, and cognitive function, were administered by Shieu et al. [47], and they reported that entering menopause one year earlier was linked with a 0.49 lower average in the composite cognitive value. In a study conducted on the medical records of 4047 women in the USA, it was reported that natural menopause before the age of 47.4 years was linked with a 19% increased risk of developing dementia [48]. Similarly, in a cohort study that was conducted with 868 women, it was reported that those who had menopause naturally under the age of 40 had worse cognitive performance in verbal fluency, psychomotor speed, visual memory, and general cognitive functions [27]. The estrogen hypothesis is one of the theories put forward to explain the connection between early menopause and a higher risk of developing dementia. Reduced brain bioenergetics and a metabolically compromised phenotype are caused by the drop in circulating estrogen levels after menopause [49]. Cognitive alterations brought on by insufficient or insufficient compensatory bioenergetic adaptation to insufficient estrogen activation not only cause the classic menopausal symptoms, but also increase the likelihood of late-onset AD in postmenopausal women. Long-term oxidative stress is increased by estrogen deprivation, which may also accelerate brain aging and worsen cognitive impairment. Beyond its important role in its reproductive capacity, estrogen also causes vasodilation, lowers LDL cholesterol levels, increases HDL cholesterol levels, and prevents osteoporosis, among other physiological actions that are as significant. Women are more susceptible to depression, osteoporosis, Parkinsonism, heart disease, and cognitive decline or dementia if their estrogen levels drop too early [36,48]. Estrogen is stored in adipose tissue because of its steroid composition. Underweight women with a BMI of 18.5 kg/m^2^ or lower are 30% more likely to experience menopause before the age of 45 compared to women with a normal BMI between 18.5 and 22.4 kg/m^2^ [50,51]. The mean BMI score in the >50-year-old menopausal group was significantly lower than in the other groups. The total cholesterol level was significantly lower in the >50-year-old menopausal group than in the other groups, and compared to the other groups, the HDL level was considerably higher. It is unclear what processes underlie the link between menopause, obesity, and cognitive abilities. It is most likely involving several interconnected pathways [52]. Increased endogenous estrogen levels have been shown to mitigate the direct or indirect effects of obesity on cognition in postmenopausal women [53,54]. When the independent relationships between the ACE-R scores and anthropometric and metabolic parameters were examined in multivariate analyses, a statistically significant, negative, and weak relationship was seen between the BMI and ACE-R scores and memory scores in the subgroups. However, Thilers et al. [55] reported that postmenopausal women in the normal BMI range (18.5–25 kg/m^2^) had a more rapid decline in measures of visuospatial ability and episodic memory than those with a BMI score >25 kg/m^2^. Moreover, all the participants had rapid postmenopausal rates of change in their verbal fluency. Obesity stimulates the production of cytokines and adipocyte-derived hormonal substances along with a proinflammatory state, which may be the key to explaining the relationship between cognitive decline and obesity [56]. Clinical studies have shown that the decrease in estrogen during the menopausal transition and postmenopause can impact brain function negatively, especially those related to verbal memory and verbal fluency [57]. In the current study, a statistically significant, negative, and weak relationship was detected between the total cholesterol level and total ACE-R score, as well as the subgroup attention-orientation, visual-spatial, verbal fluency, and memory scores. A statistically significant, weak, and negative relationship was detected between the LDL levels and total ACE-R scores and visual scores. Similar to the results herein, Tekin et al. [58] examined the relationships between the total serum cholesterol, LDL, and HDL levels and cognitive function and reported that the decrease in cognitive scores was negatively associated with high total cholesterol and LDL levels. They also reported that increased plasma HDL had protective effects on the maintenance of cognitive functions. The relationship between verbal memory and learning ability in 130 women with and without memory complaints and serum total cholesterol, triglycerides, HDL level, and LDL level was examined in a cross-sectional study by Bates et al. [59]. The HDL level was found to be significantly correlated with better verbal learning and memory performance, especially short and long immediate recall. Furthermore, they suggested that the beneficial effects of HDL on verbal memory could occur far sooner than previously thought, adding more evidence to support HDL’s involvement in normal brain aging [59]. Statistically significant, positive, and weak relationships were also detected between the HDL level and total ACE-R score and subgroup memory and verbal fluency scores in the current study. The fact that the present study was designed retrospectively, and the number of patients was limited for each group might be considered as the limitation of the study. The fact that all the cognitive and functional scores were evaluated separately in all the menopause groups and separately for all the metabolic parameters may be considered as a strength of the study.

In conclusion, menopause causes changes in hormonal and metabolic processes. The increasing role of women in social and professional life, as well as their increased life expectancy, has provided a novel perspective in terms of evaluating the cognitive and cognitive functions that menopause may cause. For this reason, broad-based prospective studies are needed to evaluate the cognitive and functional dysfunctions that might be caused by menopause.

## Figures and Tables

**Table1:** Demographic characteristics of the groups.

Variables	Patients n = 96 (100%)	Group 1 n = 32 (33.3%)	Group 2 n = 32 (33.3%)	Group 3 n = 32 (33.3%)	p
**Menopause age (years)**	44.3 ± 6.2 (35–55)	36 ± 1.4 (35–39)	44.7 ± 2.3 (40–49)	51.5 ± 1.2 (50–55)	<0.001
**BMI (kg/m** **^2^** **)**	24.2 ± 1.5 (18.2–28.5)	24.8 ± 1.3 (20.2–28.5)	24.3 ± 1.3 (19.2–27.5)	23.5 ± 1.6 (18.2–27.2)	0.003
**Education status (University)**	50 (52.1%)	20 (62.5%)	17 (53.1%)	13 (40.6%)	0.2
**Parity**	1.5 ± 0.8 (0–4)	1.5 ± 0.8 (0–4)	1.4 ± 0.8 (0–4)	1.5 ± 0.9 (0–4)	0.8
**Smoking habit**	49 (51%)	16 (50%)	16 (50%)	17 (53.1%)	0.9
**Alcohol habit**	32 (33.3%)	10 (31.3%)	14 (43.8%)	8 (25%)	0.2

* BMI: body mass index.

**Table 2:** Laboratory characteristics of the groups.

Variables	Patients n = 96 (100%)	Group 1 n = 32 (33.3%)	Group 2 n = 32 (33.3%)	Group 3 n = 32 33.3%)	p
**Fasting glucose (mg/dL)**	87.3 ± 9.5 (65–106)	90.8 ± 8.1 (75–106)	86.2 ± 10.4 (65–105)	85 ± 9 (67–102)	0.03
**Fasting insulin (mg/dL)**	82.3 ± 28.5 (11–171)	74.9 ± 22.1 (25–123)	84.2 ± 38.2 (11–171)	87.7 ± 21.4 (46–125)	0.1
**Total cholesterol (mg/dL)**	212.7 ± 22.1 (165–281)	222.7 ± 20 (186–281)	212.6 ± 20.8 (176–271)	202.8 ± 21.5 (165–262)	<0.001
**LDL (mg/dL)**	109.7 ± 21.2 (78–171)	115.3 ± 21.2 (91–171)	109.8 ± 20.7 (84–161)	104 ± 20.8 (78–155)	0.1
**HDL (mg/dL)**	51.2 ± 5.6 (36–64)	48.4 ± 4.3 (39–57)	50.8 ± 4.9 (39–63)	54.4 ± 5.9 (36–64)	<0.001
**TG (mg/dL)**	169.3 ± 17 (126–215)	174.3 ± 13.2 (155–215)	169.2 ± 17.3 (135–215)	164.3 ± 19 (126–208)	0.06
**TSH (mIU/L)**	2.13 ± 1 (2–3.9)	1.77 ± 1.12 (2–3.9)	2.37 ± 0.97 (2–3.9)	2.27 ± 0.94 (2–3.9)	0.04
**HOMA-IR**	2 ± 0.64 (2–2.7)	2.3 ± 0.17 (1.8–2.7)	1.83 ± 0.83 (2–2.7)	1.92 ± 0.63 (2–2.7)	0.005

* LDL: low-density lipoprotein, HDL: high-density lipoprotein, TSH: thyroid stimulating hormone, HOMA: homeostatic model assessment, TG: triglyceride.

**Table 3:** Comparison of scoring systems.

Variables	Patients n= 96 (100%)	Group 1 n= 32 (33.3%)	Group 2 n= 32 (33.3%)	Group 3 n=32 (33.3%)	p
**Attention orientation score**	16.5 ± 1.1 (14–18)	15.5 ± 0.9 (14–18)	16.6 ± 1 (14–18)	17.3 ± 0.7 (15–18)	<0.001
**Language score**	24.9 ± 0.7 (24–26)	24.9 ± 0.8 (24–26)	25 ± 0.7 (24–26)	24.9 ± 0.7 (24–26)	0.8
**Visual score**	14.2 ± 1.1 (11–16)	13.5 ± 1.1 (11–16)	14.2 ± 0.8 (12–15)	15 ± 0.8 (13–16)	<0.001
**Verbal fluency score**	11.1 ± 1.2 (9–14)	10.1 ± 0.8 (9–12)	11.1 ± 1 (10–14)	12.2 ± 0.8 (11–14)	<0.001
**Memory score**	21.1 ± 1.3 (18–24)	19.8 ± 0.9 (18–22)	21.3 ± 0.8 (20–23)	22.3 ± 0.8 (21–24)	<0.001
**ACE-R score**	88.1 ± 3.7 (79–84)	83.9 ± 2.2 (79–88)	88.4 ± 1.8 (95–91)	91.9 ± 1.4 (88–94)	<0.001

* ACE-R: The Addenbrooke’s cognitive evaluation battery-revised.

**Table 4:** Evaluation of the relationship between scoring systems and laboratory data.

Variables	BMI	Total cholesterol	LDL	HDL	TSH
	(r-coefficient) - p-value				
**Attention orientation score**	(−0.7) – 0.4	(−0.3) – <0.001	(−0.1) – 0.09	(0.1) – 0.06	(0.04) – 0.6
**Language score**	(−0.1) – 0.09	(−0.01) – 0.8	(−0.004) – 0.9	(0.09) – 0.3	(−0.05) – 0.5
**Visual score**	(−0.1) – 0.3	(−0.4) – <0.001	(−0.3) – 0.003	(0.1) – 0.1	(0.1) – 0.07
**Verbal fluency score**	(−0.1) – 0.2	(−0.3) – 0.002	(−0.09) – 0.3	(0.2) – 0.004	(0.1) – 0.1
**Memory score**	(−0.3) – <0.001	(−0.2) – 0.02	(−0.08) – 0.4	(0.3) – <0.001	(0.07) – 0.4
**ACE-R score**	(−0.2) – 0.01	(−0.4) – <0.001	(−0.2) – 0.04	(0.3) – <0.001	(0.1) – 0.2

* ACE-R: The Addenbrooke’s cognitive evaluation battery-revised, BMI: body mass index, LDL: low-density lipoprotein, HDL: high-density lipoprotein, TSH: thyroid stimulating hormone, HOMA: homeostatic model assessment, TG: triglyceride.
